# A Mobile App for Securely Capturing and Transferring Clinical Images to the Electronic Health Record: Description and Preliminary Usability Study

**DOI:** 10.2196/mhealth.3481

**Published:** 2015-01-02

**Authors:** Adam Landman, Srinivas Emani, Narath Carlile, David I Rosenthal, Simon Semakov, Daniel J Pallin, Eric G Poon

**Affiliations:** ^1^Brigham and Women\'s HospitalDepartment of Emergency MedicineBoston, MAUnited States; ^2^Harvard Medical SchoolBoston, MAUnited States; ^3^Brigham and Women\\\'s HospitalDepartment of MedicineBoston, MAUnited States; ^4^Brigham and Women\'s HospitalDepartment of MedicineBoston, MAUnited States; ^5^Yale School of MedicineDepartment of MedicineNew Haven, CTUnited States; ^6^US Department of Veterans AffairsWest Haven, CTUnited States; ^7^Solaris Development Inc.Newton, MAUnited States; ^8^Brigham and Women\\\'s HospitalDepartment of Emergency MedicineBoston, MAUnited States; ^9^Boston Medical CenterDepartment of MedicineBoston University School of MedicineBoston, MAUnited States

**Keywords:** mobile phone, photographs, electronic health records, telemedicine

## Abstract

**Background:**

Photographs are important tools to record, track, and communicate clinical findings. Mobile devices with high-resolution cameras are now ubiquitous, giving clinicians the opportunity to capture and share images from the bedside. However, secure and efficient ways to manage and share digital images are lacking.

**Objective:**

The aim of this study is to describe the implementation of a secure application for capturing and storing clinical images in the electronic health record (EHR), and to describe initial user experiences.

**Methods:**

We developed CliniCam, a secure Apple iOS (iPhone, iPad) application that allows for user authentication, patient selection, image capture, image annotation, and storage of images as a Portable Document Format (PDF) file in the EHR. We leveraged our organization’s enterprise service-oriented architecture to transmit the image file from CliniCam to our enterprise clinical data repository. There is no permanent storage of protected health information on the mobile device. CliniCam also required connection to our organization’s secure WiFi network. Resident physicians from emergency medicine, internal medicine, and dermatology used CliniCam in clinical practice for one month. They were then asked to complete a survey on their experience. We analyzed the survey results using descriptive statistics.

**Results:**

Twenty-eight physicians participated and 19/28 (68%) completed the survey. Of the respondents who used CliniCam, 89% found it useful or very useful for clinical practice and easy to use, and wanted to continue using the app. Respondents provided constructive feedback on location of the photos in the EHR, preferring to have photos embedded in (or linked to) clinical notes instead of storing them as separate PDFs within the EHR. Some users experienced difficulty with WiFi connectivity which was addressed by enhancing CliniCam to check for connectivity on launch.

**Conclusions:**

CliniCam was implemented successfully and found to be easy to use and useful for clinical practice. CliniCam is now available to all clinical users in our hospital, providing a secure and efficient way to capture clinical images and to insert them into the EHR. Future clinical image apps should more closely link clinical images and clinical documentation and consider enabling secure transmission over public WiFi or cellular networks.

## Introduction

### Background

Diagnosis depends on history, physical examination, and testing. Visual assessment is of paramount importance for certain conditions, such as rashes, wounds, and infections. Recording visual data has traditionally been limited to written descriptions, though digital photography is making inroads, particularly in pathology, dermatology, and plastic surgery [1]. Digital images can help us monitor changes over time, and can be shared among providers. Mobile devices with high-resolution cameras are now ubiquitous, giving clinicians the opportunity to capture and share images from the bedside [2]. However, secure and efficient ways to acquire, store, and share digital images are lacking [3].

Some clinicians already use personal mobile phones to capture patient photos. These creative clinicians email captured images to one of their email accounts, then open their email on a hospital workstation and copy and paste the images into the electronic health record (EHR). This may create privacy and safety vulnerabilities because images and patient data may remain on personal devices, and correct image-patient pairing is not assured.

We developed CliniCam, a mobile app for clinicians to securely capture images and transfer them to the EHR. We describe the application design and report usability findings from an initial group of users.

### Application Description

**Figure 1:**
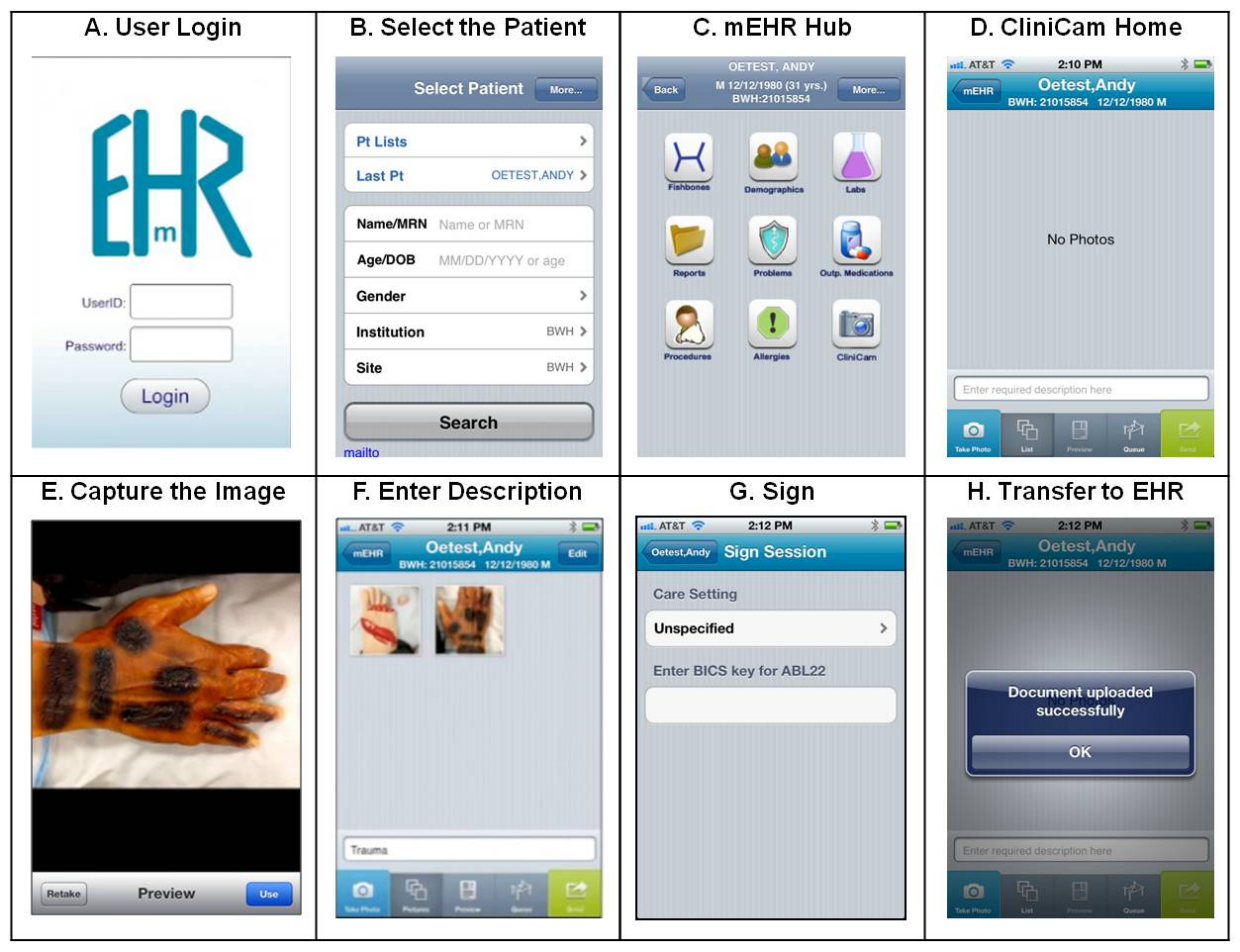
The CliniCam app. All patient and clinical information is fictitious. EHR=electronic health record; mEHR=partners mobile electronic health record app.

We developed CliniCam, an app that allows user authentication, patient selection, image capture, image annotation, and storage of the images as a Portable Document Format (PDF) file in the EHR. CliniCam is a native app for Apple iOS (iPhone, iPad) developed using Xcode (Apple Computer, Cupertino, CA). For user authentication and patient identification, we leveraged a pre-existing Partners Health Care native iOS app, mEHR, which allowed clinicians to view patients’ electronic health records remotely. Users log in to mEHR with their hospital user ID and password (Figure 1A), then search for the patient using a combination of name, medical record number, age, date of birth, and gender (Figure 1B). Users are then presented with a list of potential patient matches and select the appropriate match. From the mEHR app hub, the user then selects CliniCam from the menu of available mobile apps (Figure 1C). CliniCam is launched through a uniform resource locator (URL) scheme passing user and patient context.

The CliniCam home screen then displays, including the selected patient’s demographics in the top navigation bar (Figure 1D). The user selects “Take Photo” from the lower tab bar and then uses the camera to capture the image (Figure 1E). Up to six images can be captured per patient session; each image can be individually annotated. The image set must be labeled with a mandatory description. After the user is satisfied with the captured images and annotations/description, the user selects “Send” from the tab bar (Figure 1F). The user is then prompted to select the care setting from which the photo was taken and to enter their unique security key to sign the transaction (Figure 1G). The images are then transmitted to the patient’s EHR and a modal message box confirms the successful transfer (Figure 1H). The video in Multimedia Appendix 1 summarizes how the CliniCam app is used to capture and securely transfer clinical images to the EHR.

### Integration With the Electronic Health Record

A key feature of CliniCam is making the images available to all clinicians in the EHR. Images taken by CliniCam are viewable by all credentialed EHR users, via our institution’s clinical data repository (CDR), a system that consolidates electronic patient data from multiple systems in a single portal. Our organization has an enterprise services-oriented architecture with web services or application programming interfaces (API) that enable two-way communication with our clinical systems [4]. EHR integration of CliniCam required web services to match patients with their electronic health records and to transfer images to the EHR.

In order to link the CliniCam PDF to the appropriate patient’s medical records, the user initially searches for the patient by name and/or medical record number using patient directory services. Gender and date of birth may be used to narrow the search. Once the correct patient has been selected, CliniCam has the appropriate unique identifiers to match the photos to the patient’s EHR. Patient selection is forced before photo acquisition to ensure photos are associated with the correct patient.

To transfer the images to our EHR, we needed to select an image format that the EHR could receive. Our clinical document repository is limited to storing text or PDF documents and does not have the ability to accept images alone. In this case, CliniCam internally transforms the captured images into a PDF file, and then calls a web service to transfer the PDF to the CDR. Using the PDF format enabled us to display both the photos and metadata (patient identifiers, name of clinician capturing photos, date/time the image was captured, and annotations) in a single file. The PDF is identified as a photo document in the clinical data repository and easily available for review by any authorized clinician in our health care system.

### Security and Privacy

Ensuring the security and privacy of confidential patient information was of paramount importance in our design since the hospital and clinical users are covered entities that must comply with the Health Insurance Portability and Accountability Act (HIPAA) of 1996 [5,6]. HIPAA requires reasonable and appropriate safeguards for protecting electronic protected health information, including clinical images [7]. Despite recent calls for more clarity around HIPAA requirements for mobile health apps [8], HIPAA does not provide a list of required features and fulfillment is subject to interpretation. Therefore, we worked with our hospital’s information security officer to perform a risk analysis and to design the application with attention to access control, auditing, and encrypted data storage and network transmission.

Table 1 summarizes the features used to make CliniCam secure and HIPAA compliant. On app launch, users are authenticated using their hospital user ID and password, using Active Directory authentication. All application launches as well as patient data accesses are logged. Captured clinical images are stored in temporary files within a secure temporary storage area allocated to the app (sandbox) while the app is running, but all the data are cleared when they are uploaded to the EHR or the app is closed, so that no data are permanently stored on the mobile device. If the user is interrupted or forgets to transfer the images to the EHR, CliniCam will notify the user in 15 minutes, and will then automatically delete the clinical images 5 minutes later if no action is taken. CliniCam requires a connection to our organization’s secure WiFi network and will not function if the user is solely connected to a public WiFi or cellular network (eg, 3G or 4G LTE).

**Table 1:** Security features implemented to make CliniCam secure and Health Insurance Portability and Accountability Act (HIPAA) compliant.

Security feature	CliniCam implementation
User authentication	Requires user log-in with hospital user ID and password
Audit trail	Logs user access to patient information
Data encryption	Temporarily stores images in encrypted area of application memory
No permanent data storage	No permanent storage of patient information or clinical images. When images are transferred to the electronic health record, the images are permanently deleted from the application
Application timeout	Automatically removes clinical images from the application if not transferred to the electronic health record in 20 minutes (user receives warning notification after 15 minutes)
Secure wireless transmission	Requires connection and transmission over hospital’s secure WiFi network; data transfer over public networks is not supported

## Methods

### Study Design

We performed a preliminary usability study of CliniCam using a survey of initial app users. We sent email invitations to resident physicians from emergency medicine, ambulatory internal medicine, and dermatology to participate in the study and use the app in clinical practice for one month. Resident physicians were eligible to participate if they owned an iOS device, were willing to use their mobile device to capture clinical images, and were on clinical service during the time of the study. Clinicians responding affirmatively to the email invitation were sent instructions for installing and using the app, including a video tutorial and a link to download the app on their mobile device.

These three clinical services were selected because they commonly capture images of patients. Clinicians currently use several methods for incorporating clinical images into our EHR. Some clinicians use a digital camera to capture images, then transfer the images from the camera to a hospital workstation and embed selected images into their clinical note. Other clinicians use their personal mobile phone to capture clinical images, then email the images to their email account and copy and paste the image from their email into the EHR.

After approximately one month of clinical use, participants were emailed and asked to complete a voluntary, Web-based survey (SurveyMonkey, Palo Alto, CA; see Multimedia Appendix 2) on their experience. We analyzed the survey results using descriptive statistics.

The Partners HealthCare Human Research Committee reviewed and approved this study. Clinicians consented to participate in the study by responding affirmatively to the initial email invitation, installing the app on their mobile device, and voluntarily completing the Web-based survey. The CliniCam app was developed for routine, nonresearch use and was approved for clinical use by the hospital prior to the study. Clinicians were asked to obtain verbal consent from each patient before taking pictures per hospital policy. Patients did not participate in this research and no patient information or clinical images were reviewed or included in this usability study.

## Results

Twenty-eight resident physicians participated between July and September 2012, and the survey was completed by 19/28 (68%), with mean age 29 (53% female). They were from the following specialties: dermatology (11%), internal medicine (47%), and emergency medicine (42%). Approximately half of the respondents (53%) did not use CliniCam, many noting that they did not have a patient requiring photos during this time.

Of the respondents who used CliniCam, 8/9 (89%) found it useful or very useful for clinical practice and the same percentage wanted to continue using the application. The majority of respondents (8/9 [89%]) were also satisfied by the application’s quality of pictures and ease of use, but only 5/9 (56%) of respondents were satisfied by the application’s speed. Three of the users who did not have a clinical need for photos during the pilot indicated that they liked the CliniCam concept and hoped to use it in the future. Respondents critiqued app speed and location of photos.

Several respondents reported that the app was slow and one respondent reported losing a photo. CliniCam is designed to work only on the hospital’s secure WiFi network. Investigation of the slowness and lost photos revealed users were often connecting to other networks and that some clinical areas did not have adequate WiFi signals. To minimize these issues, we provided user education and enhanced CliniCam to check for correct WiFi network connection on launch.

Respondents preferred that clinical images be embedded (or linked) to clinical notes so they did not have to navigate to another EHR location to view the image. Some users also wanted to keep a copy of the image file separate from the EHR for teaching and research purposes.

## Discussion

### Principal Findings

We developed and implemented a mobile app that facilitates bedside photography with secure transfer into the medical record. We leveraged an existing mobile app for user authentication and patient selection, as well as an enterprise service to securely transfer images in PDF to our CDR. Participants reported that the app was easy-to-use and useful for clinical care. Users offered constructive suggestions regarding speed/network connectivity and location of clinical images within the EHR.

Two key features informed the design of CliniCam and provided value to the user and organization: (1) security features meeting HIPAA compliance, and (2) integration with the EHR. We recognized that clinicians were currently using workarounds to capture and store clinical images with their mobile devices that violated HIPAA principles. We also sought to make it easier for clinicians to automatically transfer the images to our EHR as existing workarounds were time-consuming and cumbersome. Health information technology initiatives often fail to be adopted or achieve their expected benefits because of difficulty integrating the technology with work practices [9]. In this case, adding HIPAA security features protected the patient and organization, but also had the potential to increase workload for some users. We balanced this trade-off with integration with the EHR, which reduced user workload by enabling clinicians to use their existing log-ins, to minimize manual entry of patient demographics, and to store images in the clinical document repository for easy sharing across the health care enterprise.

While planning for clinical image integration with the EHR, we discovered that our EHR does not have a predefined storage area for images or videos (similar to many current EHRs). We considered using our Picture Archiving and Communication System (PACS) that stores radiology images, but this required an electronic order be placed prior to image capture. We felt this additional step would reduce app usage and encourage workarounds. We therefore transformed captured images into a PDF, which EHRs, including ours, routinely handle. While this solution was functional, we sacrificed the ability to store and manipulate native images. Further, image storage was separate from clinical documentation. EHRs should consider enhancements to better support native clinical image storage within the EHR, and the ability to embed/link clinical images stored in external sources to EHR clinical documentation to improve image retrieval speed and efficiency.

As CliniCam and other mobile apps proliferate in health care settings [10], ensuring a robust and widely available WiFi network is critical. Given that CliniCam transfers protected health information and connects to our EHR services, we required information transmission over our secure hospital WiFi network. However, users were sometimes not connected to the secure WiFi network or did not have adequate wireless signal. Future health care provider apps should include security features enabling transmission over public WiFi and cellular networks. Hospitals should also ensure their WiFi networks have adequate coverage and bandwidth to support mobile health applications.

CliniCam is currently available to all clinicians in our hospital. Wound care nurses are documenting existing and new pressure ulcers with CliniCam [11,12], and anesthesiologists are using CliniCam to visually record Mallampati airway score during preoperative assessment. Future versions could extend this mobile platform to include video and to include other users, such as patients wishing to securely share clinical images with their health care providers [13]. Additional investigation is needed to understand CliniCam’s ability to facilitate telemedicine, specialty consultation, and reduce unnecessary health care visits [14-16].

### Comparison With Prior Work

CliniCam was conceived and developed between 2011 and 2012 when no health care provider-facing apps were available that could securely capture clinical images from mobile devices and transfer them to the EHR. At that time, we were aware of only one application, ClinPix, which allowed clinical image storage on local iOS devices with manual entry of patient demographics [17]. The most commonly reported use of digital clinical images was for store and forward teledermatology, where images were usually captured via digital camera, uploaded to a local desktop computer, and electronically transferred to a remote dermatologist’s workstation [18]. Now mobile apps are emerging in this area, but primarily support local image storage, or secure cloud-based storage, without EHR integration. Notably, Figure1 app is becoming popular and allows de-identified image sharing with medical professionals for educational purposes, but not clinical use [19]. The lack of EHR integration was a central limitation of previous products -busy clinicians were forced to access a separate system for image capture and retrieval and there was minimal ability to seamlessly share images across the care team. More recently, EHR vendors have begun to support secure image capture and transfer to the EHR in their proprietary mobile apps [20], and may benefit from the description of CliniCam and usability findings reported here.

### Limitations

There are several limitations to this study including the limitation to generalize the results, small convenience sample, and subjective outcomes.

CliniCam was designed for one hospital’s EHR, extending an existing mobile app and leveraging services. The application works only on Apple iOS systems, and other tools would be needed for clinicians using other mobile devices. However, the app design is broadly generalizable. EHR vendors could add similar functionality to their own mobile applications [20] or expose web services (APIs) allowing 3^rd^ party software developers to provide secure clinical image capture functionality. Also, if vendors support the Substitutability Medical Applications, Reusable Technology (SMART) platform, a single version of CliniCam could be created on the SMART architecture to work across all EHR systems [21]. For institutions without access to an existing mobile EHR application and/or enterprise services, a standalone version of CliniCam could be developed with image storage in HIPAA-compliant cloud storage.

In this report, we describe the application and provide usability results from initial users. The small convenience sample may not be representative of all physicians and could be biased towards more technically savvy physicians overstating the app’s ease of use. Furthermore, the results may be biased because many pilot users did not use CliniCam during the pilot -they may not have appreciated the potential value of an image capture tool or they may not have had a need for such a tool. An array of sociotechnical factors can influence a clinician’s use of health information technology (HIT); including hardware and software, clinical content, human computer interface, people, workflow and communication, organizational policies and procedures, external rules, regulations, pressures, and system measurement and monitoring [22]. In this case, some potential users may not have had mobile devices available at the point of care and others may have found that CliniCam did not fit with their workflow [9]. Previous studies have been devoted to understanding the barriers and facilitators of HIT use [23,24]; a future study could similarly investigate the sociotechnical factors influencing CliniCam adoption [23,24].

A larger sample would be needed for generation of statistically robust measures of usability and satisfaction. Furthermore, our measures of app usefulness were based on a 5-point, subjective Likert scale, not process or clinical outcomes. Demonstrating hard outcome benefit would require large samples and the value of the tool seems self-evident; therefore, we suggest future work focus on improving usability and clinical workflow.

### Conclusions

We created a secure and convenient mobile application for acquiring digital images and storing them in our EHR that is now available for all clinical users in our hospital. Implementation went well, though network connectivity limitations were an important shortcoming. Combining these reported experiences with mobile development tools and EHR services, other institutions and vendors should be able to implement secure image sharing applications.

## Multimedia Appendix 1

CliniCam video.

## Multimedia Appendix 2

CliniCam survey instrument.

## Abbreviations

API: application programming interface
App: application
CDR: clinical data repository
EHR: electronic health record
HIPAA: Health Insurance Portability and Accountability Act
HIT: health information technology
PACS: picture archiving and communication system
PDF: portable document format
SMART: substitutability medical applications, reusable technology
