# Supplementary material for: A Mobile App for Securely Capturing and Transferring Clinical Images to the Electronic Health Record: Description and Preliminary Usability Study
Source: JMIR Mhealth Uhealth. 2015 Jan 2;3(1):e1. doi: 10.2196/mhealth.3481 (PMC4296096; doi:10.2196/mhealth.3481)
Supplement: Supplementary file 2 [file mhealth_v2i4e59_app2.pdf]

CliniCam Satisfaction Survey

Introduction

Thank you for participating in the CliniCam mobile application pilot study. We are interested in your experiences with CliniCam and invite you to participate in this survey. Your feedback will help BWH and Partners determine whether or not to make this mobile application available to all clinicians. In addition, you will also help determine if there is additional commercial opportunity for this application beyond Partners Healthcare. We would greatly appreciate your feedback.

This survey should take less than 5 minutes to complete.

Your participation is voluntary. If you have questions about the survey, please contact Dr. Sri Emani, co-investigator of the study, at semani1@partners.org. If you have any questions about the CliniCam app, please contact Dr. Adam Landman at alandman@partners.org. If you would like to talk with someone not involved in this research, please contact the Partners Institutional Review Board at (617) 424-4100.

Demographics

1. Age:

2. Gender:

Choose from drop-down menu

3. Specialty:

Choose from drop-down menu

CliniCam Satisfaction Survey

4. Position:

Choose from drop-down menu

Main Survey

5. For approximately how many unique patients have you used the CliniCam app to take clinical pictures?

- ◯ None
- ◯ 1 - 10
- ◯ 11 - 20
- ◯ > 20

6. Please explain why you did not use CliniCam to take clinical pictures:

7. Do you have any other comments on the CliniCam app? (Please list them below)

8. On average, how many pictures did you take for each patient?

- ◯ 1 - 10
- ◯ 11 - 20
- ◯ > 20

CliniCam Satisfaction Survey

9. Please rate your satisfaction with the CliniCam app:

	Very Satisfied	Somewhat Satisfied	Neutral	Somewhat Dissatisfied	Very Dissatisfied
Ease of use	◯	◯	◯	◯	◯
Quality of the pictures	◯	◯	◯	◯	◯
Speed	◯	◯	◯	◯	◯
Overall satisfaction	◯	◯	◯	◯	◯

10. If you have comments on ease of use, quality of pictures, or speed, please enter them below:

11. Please rate your satisfaction with these features of the CliniCam App:

	Very Satisfied	Somewhat Satisfied	Neutral	Somewhat Dissatisfied	Very Dissatisfied
Annotation of Pictures	◯	◯	◯	◯	◯
Ability to Take Multiple Pictures	◯	◯	◯	◯	◯
Description of Set of Pictures (Title)	◯	◯	◯	◯	◯
Storage as PDF File	◯	◯	◯	◯	◯
Storage in Document Repository (i.e. LMR --> Results --> Big Board --> Notes)	◯	◯	◯	◯	◯

12. If you have any comments on features of the CliniCam app please enter them below:

13. Are there other features you would like to see supported in future versions of the CliniCam app?

◯ No

◯ Yes

CliniCam Satisfaction Survey

14. Please explain other features you would like to see supported in future versions of the cliniCam app:

15. Overall, how useful do you find the CliniCam app in your clinical practice?

Very Useful	Useful	Neutral	Not Useful	Not at all Useful
◯	◯	◯	◯	◯

16. I would like to continue using this app to capture digital images

Strongly Agree	Agree	Neutral	Disagree	Strongly Disagree
◯	◯	◯	◯	◯

17. Do you have any other comments on the CliniCam app? (Please list them below)
